# Food searches and guiding structures in North African desert ants, *Cataglyphis*

**DOI:** 10.1007/s00359-015-0985-8

**Published:** 2015-02-08

**Authors:** Siegfried Bolek, Harald Wolf

**Affiliations:** Institute of Neurobiology, University of Ulm, 89069 Ulm, Germany

**Keywords:** North African desert ant, *Cataglyphis fortis*, Navigation, Food search strategy, Extended landmark orientation

## Abstract

North African desert ants, *Cataglyphis fortis,* use path integration as their primary means of navigation. The ants also use landmarks when these are available to improve navigation accuracy. Extended landmarks, such as walls and channels, may serve further functions, for example, local guidance or triggering of local vectors. The roles of such structures were usually examined in homing animals but not during food searches. When searching for familiar feeding sites, *Cataglyphis* may show intriguing deviations from expected search performances. These may result from the presence of extended landmarks, namely experimental channels. Here we scrutinise this hypothesis of landmark guidance in food searches. We prevented the ants from seeing the channel walls by covering their eyes, except the dorsal rim area. This experiment was repeated in the open test field with an alley of black cylinders to extend our findings to a more normal foraging environment. Ants with covered eyes did not deviate from expected search performances, whereas ants with normal eyes extended their searches along the axis of the leading structures by 15–20 %, in both channels and landmark alleys. This demonstrates that *Cataglyphis* orients along extended landmarks when searching for familiar food sources and alters its search pattern accordingly.

## Introduction

*Cataglyphis fortis* (Wehner 1983) ants live in the salt flats of North Africa. Due to yearly winter flooding, these sabkhas are level, bare and virtually devoid of landmarks. In this environment *C. fortis* ants rely on vector navigation as their primary means of navigation. Foragers readily accept additional orientation cues when navigating their environment, however, such as visual landmarks (Collett et al. 1992; Collett 2010, 2012; reviews in Collett and Collett 2002; Wehner 2009) as well as tactile (Seidl and Wehner 2006), chemical (Steck et al. 2009) and magnetic or vibration cues (Buehlmann et al. 2012). Indications for the use of such cues were already described 100 years ago in the closely related species *Cataglyphis bicolor* (Santschi 1913). Felix Santschi reported that experienced workers of *C. bicolor* follow habitual routes through the vegetation. Since *Cataglyphis* species do not use pheromone trails, these routes have to be defined by other means, namely by visual structures along the trail. These visual cues are employed for navigation in North African *Cataglyphis* species, as well as in Australian *Melophorus bagoti*, in South African *Ocymyrmex* and indeed in other hymenopterans (Collett and Rees 1997; Collett et al. 2002, 2007; Zeil et al. 2003; Collett and Collett 2002, 2009; Cheng et al. 2009; Graham and Cheng 2009; Wehner 2009; Collett 2010, 2012; Philippides et al. 2011). Here we present experiments suggesting that *Cataglyphis* also uses channel-like structures and extended landmark arrays in navigation towards and particularly in search for familiar food sources.

The weighting of the two navigation mechanisms, path integration and landmark orientation, depends primarily on the state of the path integrator in *Cataglyphis* ants (Bregy et al. 2008). Far away from the nest, at the beginning of a return travel after finding food, path integration is the dominant means of navigation. In the course of the homing trajectory, landmark-based navigation gains influence, and close to the nest landmark cues may become the dominant means of navigation (e.g., Bregy et al. 2008; see also Müller and Wehner 2010 for the South African desert ant *Ocymyrmex*). In addition to the state of the home vector, familiarity with landmarks and panoramic views and context may play a role in how different cues are weighed in navigation (Wolf and Wehner 2000; Collett et al. 2001; Collett and Collett 2002, 2009; Bisch-Knaden and Wehner 2003; Narendra et al. 2007; Müller and Wehner 2010). In the Australian desert ant *Melophorus bagoti* that typically inhabits more cluttered environments, orientation with regard to panoramic views and landmarks typically dominates vector navigation (Kohler and Wehner 2005; Narendra 2007; Schultheiss and Cheng 2013; Cheng et al. 2006, 2014).

**Fig. 1:**
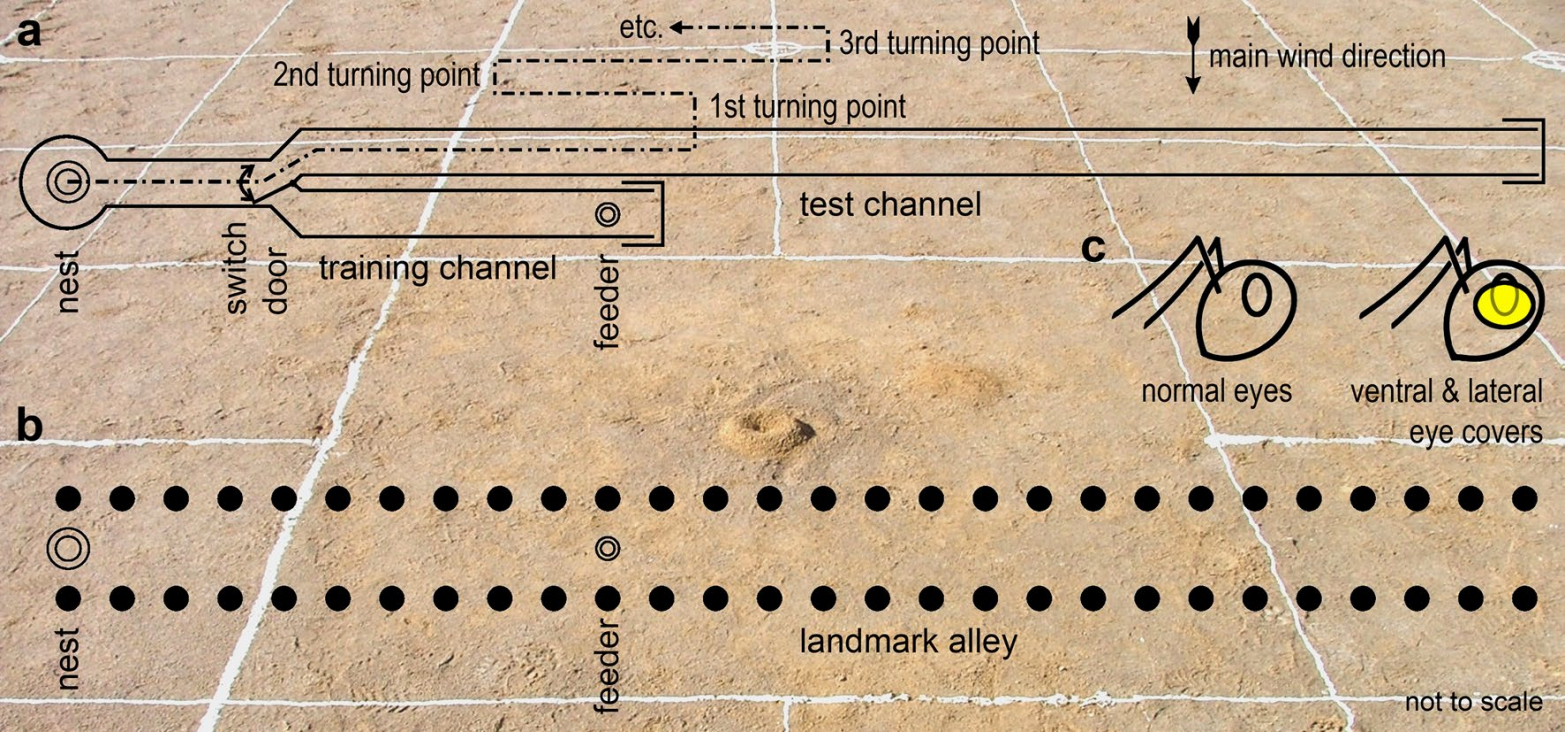
Outlines of the experimental set-ups. **a** Channel experiments. Nest and feeder were connected by a training channel; a test channel was arranged upwind in *parallel* and close to the training channel. Experimental animals were guided into either test or training channel by a switch door. Above the test channel, desert ants search behaviour around the assumed feeder position is illustrated schematically. The initial three turning points are indicated; the initial six turning points were evaluated, or just the first turning point. **b** Field experiments. The alley of *black* cylinder landmarks is shown as it extends from the nest entrance to the feeding site and 17 m further. Please note that the diagrams in **a** and **b** are not to scale, e.g. the nest-feeder axis is strongly compressed in **b** to accommodate the landmark alley of 27 m length, with only 0.5 m width; landmark alley is drawn to scale in Fig. 5a. **c** *Icons* used in the following figures to indicate experimental groups that were not manipulated and had normal eyes (*left*) and ants that had their ventral and lateral eye parts covered with opaque paint (*right*). The background picture shows a nest entrance, with a white 2 × 2 m recording grid painted on the desert floor

**Fig. 2:**
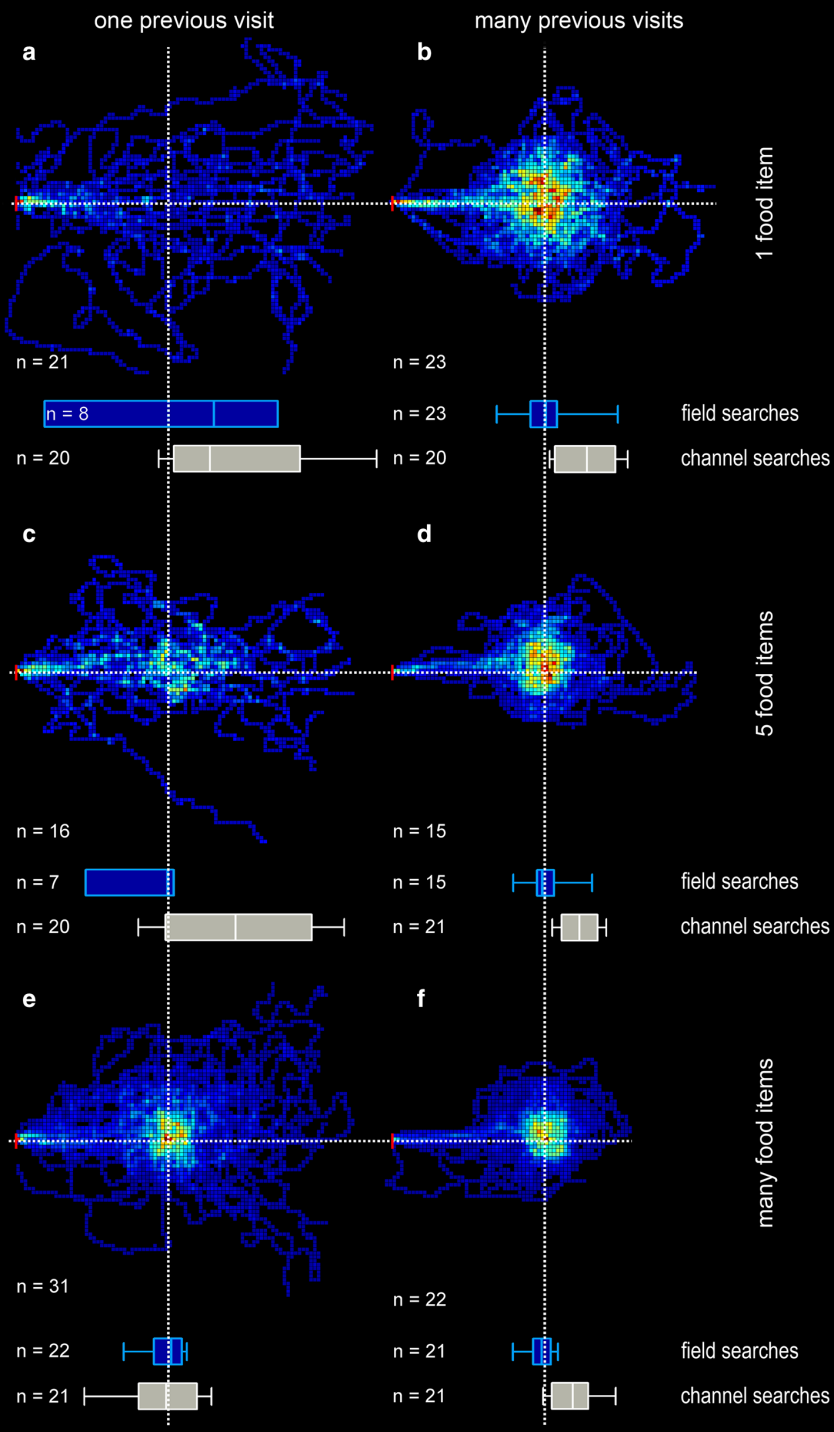
Desert ant food search behaviour is dependent on (1) the amount of food present in the feeder, (2) the experience during repeated visits, that is, learning about feeder reliability and (3) the experimental situation, that is, channel or field experiments. *Field searches* In experiments on the open desert terrain, the number of ants’ visits to each 25 × 25 cm pixel of the density plot was recorded, summed and normalised to the maximum number of visits per pixel observed in the plot. The *darkest red* represents the highest density (100 %), the *darkest blue* just a single visit (note individual walking trajectories discernible close to the plot margins), and *black* areas were not searched at all (0 %). Nest-feeder distance was 10 m; nest position is noted by *red indicator* *line* on the *left* of each graph. Feeder position before testing is marked by *white dotted cross lines*; food supply of the feeder and number of previous (training) visits are noted in the margins of columns and rows, respectively; numbers of ants tested are noted in the lower *left* of each plot. *Field searches along nest-feeder axis* Search performances along the nest-feeder axis are shown as *blue* *box*-and-*whisker* plots just below the density plots they are derived from (see “Methods”; any movements perpendicular to the nest-feeder axis were disregarded, and six turning points on the nest-feeder axis were evaluated). *Channel searches* Corresponding *grey* box-and-*whisker* plots from channel experiments are shown below the plots derived from the field experiments. Data of the channel experiments are taken from a previous study (Bolek et al. 2012b); see “Methods”. *Box*-and-*whisker* plots show medians, 25 and 75 % percentiles (*box* margins) and 10 and 90 % percentiles (*whiskers*) in this and all following figures

When examining food search behaviour in *C. fortis* in channel experiments in previous studies (Figs. 1a, 2, grey box-and-whisker plots) (see also Wolf et al. 2012), experienced foragers showed a notable and consistent shift of their search behaviour towards the far end of the channel (Bolek et al. 2012b), that is, well past the actual feeder position they had visited previously. This shift was reliable and consistent, and it occurred only in experienced ants that had foraged several times at the experimental feeding station (Fig. 2b, d, f; grey box-and-whisker plots; details in “Results”). Naïve ants that had visited the feeder just once, by contrast, accurately centred their search on the previous feeder position (Fig. 2e), except when only a single food item had been offered, and removed by the ant, on that first visit (Fig. 2a). Considering the use of landmark and panorama orientation outlined above, we hypothesised that this shift in the searches of experienced ants is due to the use of the channel walls as visual leading structures that guide the ants to the feeder location. In this scenario, the shift in an ant’s search centre is due to the use of two competing navigation cues. One cue is path integration, centring the search on the familiar feeder position; the other cue is the channel walls guiding the search along the channel without a defined distance, thus extending the search past the feeder position (see similar line of argument for *Melophorus* in Schwarz et al. 2012).

Here we examine this hypothesis in more detail with two main objectives in mind. First, we strive for a better understanding of food search behaviour that has been studied much less than nest search strategies (Bolek et al. 2012b; Schultheiss and Cheng 2013; Schwarz et al. 2012). Second, we want to scrutinise the role of extended landmarks in shaping search behaviour. This latter aspect may bear on nest search behaviour, too, although certainly to a smaller extent than in the case of food searches.

## Materials and methods

Experiments were performed between June and September in 2010 and 2011, in salt flats near the Tunisian coastal village Maharès (34.53N, 10.55E WGS84). All foragers from a given experimental nest were marked with individual colour codes using car paint. Only naïve *Cataglyphis fortis* (Forel 1902) (Wehner 1983) were used for experiments, with the term naïve concerning the feeder location and its surroundings. The animals were killed after they had completed the experiment.

**Fig. 3:**
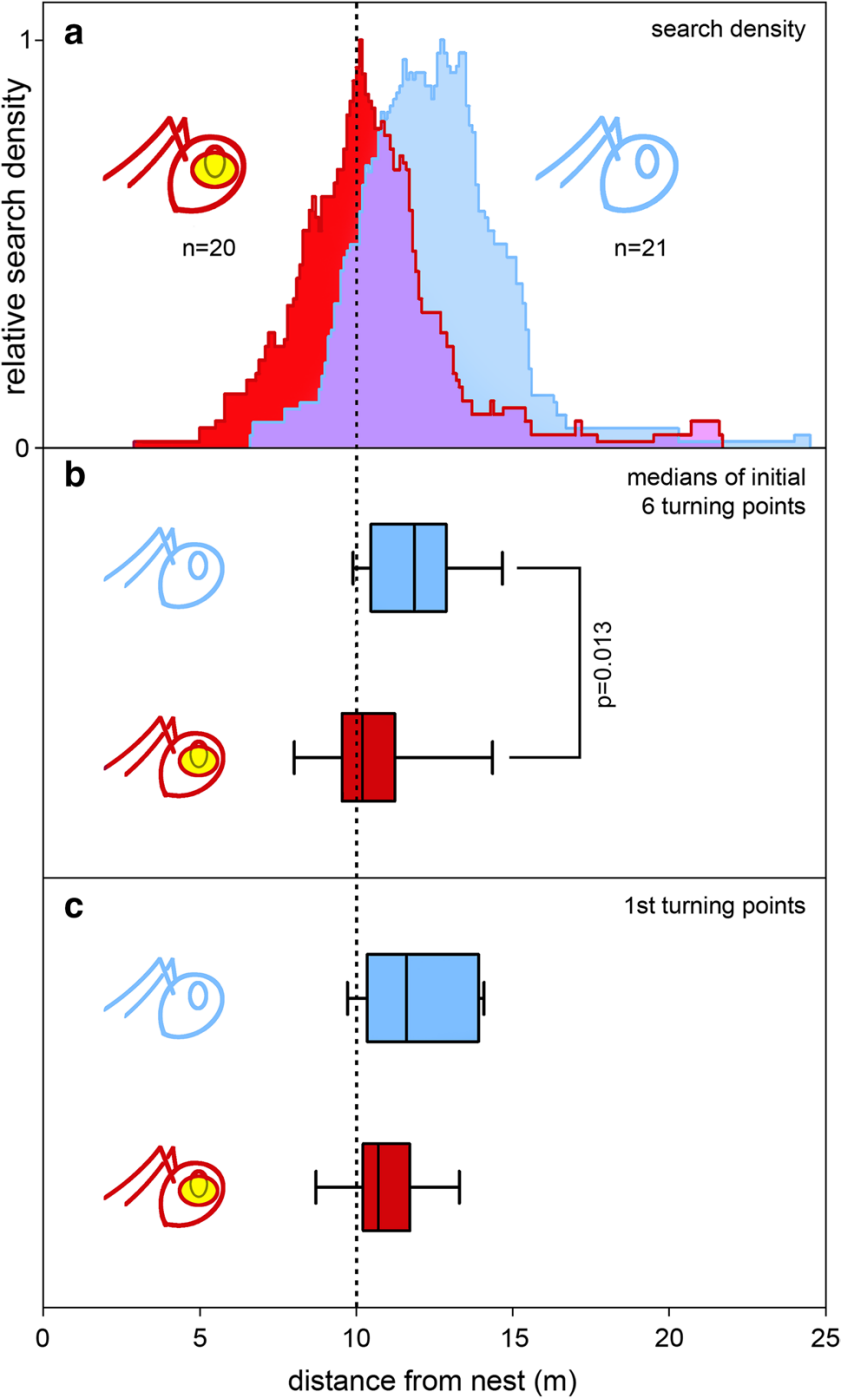
Search performances of desert ants in channel experiments. **a** Search distributions of ants trained and tested in channels. *Dashed line* indicates the training distance of 10 m. **b** Ants that were trained and tested without eye manipulations (channel control group) had a median search centre at 11.85 m with a spread of +0.99 m away from and −1.34 m towards the nest (75 and 25 % percentiles, respectively, in this and all following figures). Data for ants without eye covers were taken from a previous study ((Bolek et al. 2012b); see “Methods”). For ants with covered ventral and lateral eyes (channel “blind” group), the respective values were 10.20 m (+0.95, −0.55 m). Significant difference is indicated by a *bracket* connecting the channel control and channel “blind” *box* plots, and significance levels are noted next to the *bracket* (in this, like all following figures). **c** Only the first turning point (see Fig. 1a) was evaluated for this *box* plot, other details as in **b**. The channel control group had a median of the first turning points at 11.60 m (+2.30 and −1.22 m); for the channel “blind” group, the respective values were 10.70 and +1.00, −0.45 m

Feeding sites were established 10 m from the ant nests, consisting of petri dishes 32 mm in diameter and levelled into the ground. The feeder was filled with food crumbs: Biscuits of the Tunisian brand Saida (Sotubi Bisquiteria, Megrine, Tunisia) were cut and sieved to approximately 1.5 by 1.5 mm and flavoured over night with a few drops of mango juice. In most experiments, the feeder was filled with a large amount of food crumbs, more than 800 pieces. To distinguish shifts in search behaviour for plentiful feeding sites (Bolek et al. 2012b) from effects of sector fidelity after finding just single food items (Wehner et al. 1983; Schmid-Hempel 1984), we also offered one and five food items in the feeder (Fig. 2; details in legend). This also allows comparison to previous data from channel experiments suggesting a shift in food search behaviour with experience (Bolek et al. 2012a, b; Wolf et al. 2012). Data for channel experiments without eye covers were taken from a previous study (Bolek et al. 2012b). These data are presented as the lower, grey box-and-whisker plots in Fig. 2 and as the group without eye covers in Fig. 3.

Two types of experiments were carried out, channel experiments and field experiments, the latter providing a more normal foraging environment for the ants. The set-up for the channel experiments consisted of two parallel channels (U-shaped aluminium profiles, 7 cm wide, 7 cm high) that were connected to the nest via a switch door (Fig. 1a). One channel, the training channel, contained a feeder filled with biscuit crumbs at a distance of 10 m from the nest entrance (while extending to a total length of at least 11.5 m from the nest entrance). The other channel, the test channel, contained no food. It was located on the upwind side of the training channel to prevent food odours from being blown into the channel and distract the searching ants. The test channel extended to about 35.5 m from the nest, thus avoiding that the searching foragers reached the end of the channel. By means of the switch door, individual ants could be guided selectively into either training or test channels.

Extension of the channels well past the feeding site minimised the possibility that the ants used the end of the channel as a landmark. The divergence angle of the ommatidia in the *Cataglyphis* eye is between 3° and 7° (Eheim and Wehner 1972; Zollikofer et al. 1995), and the acceptance angle is at least twice as large. Altogether, resolution of the *Cataglyphis bicolor* eye is about 7° in the frontal eye area (Eheim and Wehner 1972), and certainly worse in the smaller *Cataglyphis fortis* studied here (Räber 1979). This makes the channel end too small to be resolved as a landmark beyond distances of 133 and 67 cm, for 3° and 7° resolution, respectively. The training channel extended for at least 150 cm beyond the feeder, and it was closed at the end to avoid contrasts between channel walls and an open channel end. We also made every effort to make test and training channels as similar as possible, including the random exchange of channel segments on a daily basis. This should avoid any learning of visual features of the channels.

Three experimental groups were examined in the channel experiments. The first group was a novice group that had visited the feeder only once before being tested (channel novice group). The ants in the following groups had foraged at least five times at the feeder before being tested; they were thus considered to be trained in the sense of being familiar with the feeder and the training situation. The second group was a control group (channel control group) that was trained and tested without manipulations. Data for channel novice and channel control groups were recorded in the course of a previous study (see above Bolek et al. 2012b). The third group was the test group (channel “blind” group) and had the ventral and lateral parts of their eyes covered with car paint in both training and test situations. Eye covers were applied under a dissection microscope. The paint covered the ventral and lateral parts of the eyes, leaving only the most dorsal part open, including the dorsal rim area, a region used by the animals for their sky compass (review in Wehner and Labhart 2006). We put “blind” into quotation marks to indicate that the eyes were not completely covered, though certainly the lateral parts necessary for landmark recognition (Wehner et al. 1996). Eye covers were checked after the ants had been tested, and data from ants without intact eye covers were discarded.

Search trajectories in the test channel were recorded to the nearest 0.1 m by means of a measuring tape strung along the test channel. We recorded the first six turning points of the ants, a turn being defined as a U-turn followed by at least 40 cm locomotion in the new direction. From these six turning points, the median was calculated as the search centre of each ant. These search centres were then used as data for further evaluation, statistical analyses and construction of diagrams (Sigma Plot 9.01 with Sigma Stat 3.11 integration; both Systat Software, Inc. San Jose, California, USA).

Field experiments were carried out on a flood plain devoid of any landmarks or conspicuous visual panorama. A 20 by 20 m grid (line spacing 2 m) was painted on the ground adjacent to a nest with diluted white wall paint (Fig. 1, background). At the centre of this grid, 10 m from the nest entrance, a feeding site was established (Fig. 1b). The feeder was levelled into the soil to fit evenly with the desert surface and was thus invisible from the approaching ants’ point of view.

As in the channel experiments, training in the field experiments started when an ant encountered the feeding site by chance. After the animal had paid at least five foraging visits to the feeding site, it was considered to be trained and the feeder was removed. The next foraging run of the ant was recorded on grid paper for 2 min and 30 s (two ants had to be excluded from further evaluation since they had returned to the nest earlier). No remnants of food or food odour were left on the test field because the feeder had been placed in a larger petri dish that collected any food crumbs dropped by ants during selection of a proper item from the feeder. This larger petri dish was removed for testing together with the feeder. The search paths of the ants revealed no signs of ants noticing the previous feeding site, even when their paths crossed the previous feeder location. To distinguish shifts in search behaviour brought about by the walls in the channel experiments from effects of sector fidelity reported previously (Wehner et al. 1983; Schmid-Hempel 1984), we also recorded the searches of a set of ants in the open terrain that had visited the feeder only once before being tested (Fig. 2; details in legend). These recordings also served for comparison with previously published data (Bolek et al. 2012b) that had reported a shift in food search behaviour with experience and with different amounts of food in channel experiments (above Bolek et al. 2012b; Wolf et al. 2012).

Three experimental groups were tested in the field experiments. The first group was a control group (field control group), consisting of ants that were not manipulated and were trained and tested on the plain field. For the second and third groups, an alley of landmarks was arranged on the field. The landmarks were black cylinders, 10 cm wide and 15 cm high. To form the alley, the landmarks were placed along the line connecting nest and feeder. A pair of landmarks was placed at every metre, with a gap of 50 cm between the two lines of landmarks. The alley started at the nest entrance and stretched past the feeding site (at 10 m) to a total length of 27 m (Fig. 1b). The second group was trained and tested with the alley present but without any further manipulations (field visual group). The third group had the ventral and lateral parts of their eyes covered, as described for the channel “blind” group, and was trained and tested with the alley present. These ants formed the field “blind” group.

For data analysis, the recorded search trajectories were digitised using a graphic tablet (Wacom Intuos 3, Wacom Europe GmbH, Krefeld, Germany). Search runs were digitised and analysed, and graphic diagrams were constructed using custom-written scripts in MATLAB R2010b (The MathWorks, Inc. Natick, Massachusetts, USA). Two major evaluations were performed with the field runs. The “distance search centre” was calculated as the value corresponding to the search centre in the channel experiments. This was done by considering only movements in the nest-feeder direction and disregarding movements perpendicular to the nest-feeder axis (thus getting one-dimensional data as recorded in the channels). The same criterion as in the channel experiments was applied to identify turning points (above). The first six turning points were extracted, and their median was used as the distance search centre. Animals that did not yield six turning points were disregarded for this evaluation (note reduced animal number especially in Fig. 2a, b, and in the box-and-whisker plots in Fig. 2, upper, blue plots in each figure part). In the two-dimensional plane, search runs were analysed by search area, search centre, search width and search length. According to Merkle and Wehner (Merkle and Wehner 2010), the search area was calculated by multiplying the length of the search (total range in nest-feeder direction) with the width of the search (range orthogonal to nest-feeder axis). The search centre was defined as the median of all *x* and *y* values of a run. For the analyses in the two-dimensional plane, only the search portions of the ants’ trajectories were used, disregarding the ants’ approach to the feeding site. Evaluation thus started after the point where the run changed its direction by at least 30° and did not revert to the former direction for at last another 3 m. This criterion to distinguish between the behaviours “following a vector” and “searching for a site” has been used repeatedly in studies of home search behaviour (Merkle et al. 2006; Merkle and Wehner 2008, 2010).

## Results

### Sector fidelity and point fidelity

Figure 2 gives a comprehensive overview of food searches in desert ants and illustrates how search behaviour is shaped by experience with a feeding site, by food abundance and by the experimental situation. Two features of the search density plots are of particular interest here. First, the ants change their search behaviour from a mode termed sector fidelity (Fig. 2a; Wehner et al. 1983; Schmid-Hempel 1984) towards a mode recently described as point fidelity (Fig. 2f; Wolf et al. 2012) when searching in the open desert terrain. Ants that had visited the feeder only once and had encountered (and presently removed) just one food item tended to walk past the feeder position without notice (Fig. 2a), or with little notice if they had encountered five food crumbs (Fig. 2c). They searched the previously successful section of the nest surrounds at no specific distance, hence the term sector fidelity. Sector fidelity appears to represent the normal mode of foraging for isolated prey items such as arthropod carcasses scattered across the desert. It is important to consider sector fidelity here since it may resemble the distance deviations observed in focussed searches, at least in plots that evaluate search densities in channels (Bolek et al. 2012b).

Focussed searches were observed in ants that had encountered food reliably at the feeding site on several subsequent occasions (Fig. 2b, d, f), or that were offered a large amount of food (>800 biscuit crumbs) on their first visit (Fig. 2e). These point-fidelity searches differ from sector fidelity searches by exhibiting concentric search distributions with a central peak. This is true for the open field, while in channel experiments point-fidelity searches exhibit a clear maximum at a particular position along the channel (Bolek et al. 2012a). Figure 2c shows an intermediate situation where five food items were offered initially, and upon their next visit, the ants’ searches exhibited a slight concentration around the feeder position while still extending well past that position after an initial check of the previous feeder location, in accord with sector fidelity. Point fidelity is illustrated by the experienced foragers in Fig. 2b, d and f.

Another feature notable in Fig. 2 is the outbound paths leading from the nest into the vicinity of the previous feeder position. These paths become straighter and are more clearly directed towards the feeder position with increasing experience (compare left and right sets of recordings in Fig. 2). This aspect was not further evaluated here in view of previous quantitative analyses (Wolf 2008).

### Food searches with shifted search centres in channel but not in field experiments

The second notable feature in Fig. 2 is a distal shift of the search centres in experienced ants that searched for plentiful food sources in experimental channels (grey box-and-whisker plots at the bottoms of each figure part). This was true in particular when the feeder had been equipped with many food items (compare grey bottom plots in Fig. 2e, f). A shift was not discernible in *novice* foragers returning to a plentiful feeder (Fig. 2e). Novice foragers that had encountered and retrieved just single food items during their previous visits (grey bottom plot in Fig. 2b) appeared to exhibit a similar shift. However, this shift most probably is an artefact of the evaluation procedure and actually represents sector fidelity searches (below). This is indicated by the large scatter of the search distribution and by comparison with the corresponding searches in the open-field situation (blue bottom plot in Fig. 2a). Novice foragers revisiting a feeder previously equipped with five food items appear to represent an intermediate situation (Fig. 2c).

We first scrutinised whether or not this shift in search centre is a particularity of channel experiments, according to our hypothesis that the shift is produced by the channel walls acting as leading structures (see “Introduction”). We thus recorded food searches in the open desert terrain with conditions otherwise similar to the channel searches. In this situation, no shifts in search centres occurred, and especially not in experienced ants that were familiar with the feeding site from several previous visits (Fig. 2b, d, f; blue, upper box-and-whisker plots). In the cases where just one or five food items had been offered to the ants on their only previous visit to the feeder (Fig. 2a, c), they exhibited no focussed searches (Fig. 2a), or paid minimal attention to the previous feeder position (Fig. 2c). This behaviour was described previously as sector fidelity (Wehner et al. 1983; Schmid-Hempel 1984). The position of the median in the box-and-whisker plot in Fig. 2a (blue, upper box plot) is more than 3 m past the nest-feeder distance. The ants’ search trajectories do not show any focus on the previous feeder position (Fig. 2a, top density plot), however, and the interquartile range is much larger than in any of the other plots. This demonstrates that the median value does not represent a search focus but rather an artefact of the evaluation of the animals’ “turning points” in projections of the walking trajectories on the nest-feeder axis (see “Methods”). Finally, six “turning points” were not often recorded along the nest-feeder axis in ants that did not exhibit focussed searches, which is reflected in the strongly reduced animal numbers in Fig. 2a, c (*n* = 8 and *n* = 7 in blue, upper box plots, compared to *n* = 21 and *n* = 16 in the corresponding two-dimensional search density plots).

The corresponding searches in channels (Fig. 2, grey box-and-whisker plots at the bottom of each figure part) exhibited distinct and consistent shifts in a direction away from the nest in experienced ants. This is particularly obvious when the channel searches are compared to box-and-whisker plots constructed from the field searches by evaluating only search components along the nest-feeder axis, and considering six turning points identified on this axis to generate data comparable to those from the channel searches (Fig. 2, blue box-and-whisker plot just below each field density plot).

The fact that no shift in search centres occurred in the open-field experiments appeared to support our hypothesis that the channel walls act as visual cues and thus are responsible for the observed shift. We therefore scrutinised the role of visual input further.

### Experiments in channels—elimination of visual input abolishes search shift

The direct comparison of untrained and trained ants (with intact eyes) visiting a full feeder revealed a significant difference between the search medians of these two groups (*p* = 0.003, *t* test). In ants that had foraged only once before being tested (channel novice group), the search centre was at 9.85 m (*n* = 21). In ants that had paid at least five visits to the feeder (trained group; channel control group), the search centre was at 11.85 m (*n* = 21) (Fig. 2e, f; grey, lower box plots; data from Bolek et al. 2012b), that is, almost 2 m, or 20 %, past the previous position of the feeder. To examine possible reasons for this shift in search behaviour, in all following groups, the ants had foraged at least five times at the feeder before they were tested. This procedure also ascertained that the ants indeed searched for the feeder (Fig. 3), rather than exhibited sector fidelity and just walked past the feeding site to explore the channel further (like in Fig. 2a, and partly in Fig. 2c). In ants with covered ventral and lateral eyes (channel “blind” group), the search centre was at 10.20 m (*n* = 20). This channel “blind” group was thus significantly different (*p* = 0.013) from the channel control group (Fig. 3a, b).

Very similar results were obtained when considering just the initial (first) turning point of each ant, rather than the median of the initial six turning points (Fig. 3c). The first turning point is usually considered to reflect the vector length in path integration, while the median of subsequent turning points characterises the search centre (e.g. Cheng and Wehner 2002; Narendra et al. 2007, 2008)—which might differ from the vector as determined by the first turning point (Cheng and Wehner 2002). The medians of the first turning points and of the searches constructed from the initial six turning points had similar values in our experiments, however (Fig. 3b, c). The channel groups with and without eye covers were not significantly different, probably due to larger scatter.

### Leading structures in the open field

**Fig. 4:**
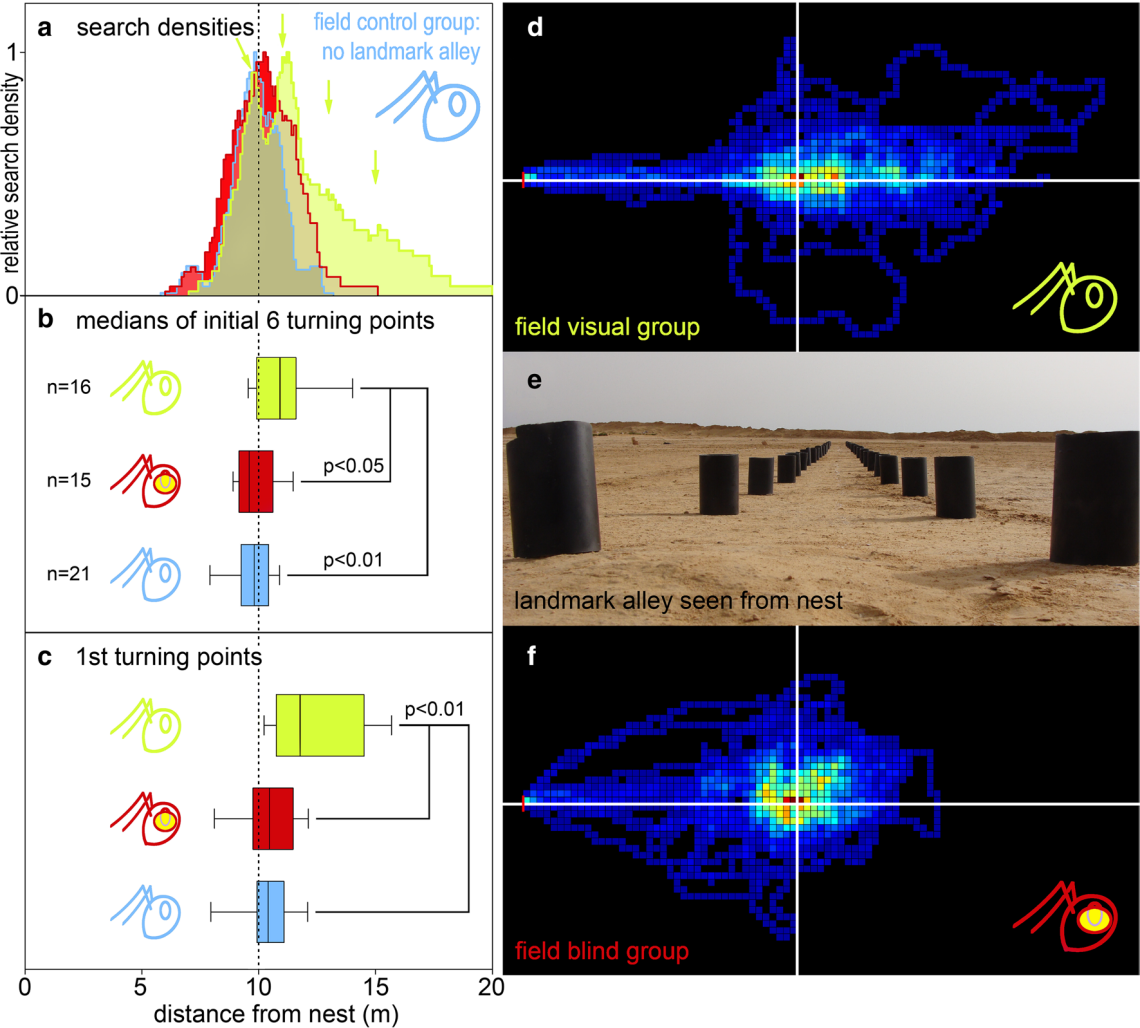
Search distributions of ants in field experiments. **a** Only movement in the nest-feeder axis was evaluated to extract turning points comparable to those in the channel experiments (see “Methods” and legend Fig. 2) and to construct the density plots shown. *Arrows* point to peaks and shoulders in the search distribution of the field visual group;* arrow *positions are at 10, 11, 13 and 15 m. **b** Corresponding *box*-and-*whisker* plots. With the alley of landmarks present, ants without manipulations (*green*, field visual group) had a distance search centre at 10.90 m (+0.65 m; −0.98 m) and ants that had their ventral and lateral eyes covered (*red*, field “blind” group) had their distance search centre at 9.59 m (+0.96 m; −0.4 m). Ants that were trained and tested without landmark alley and without eye manipulations (*blue*, field control group) had their distance search centre at 9.81 m (+0.61 m; −0.51 m). **c** Only the first turning points were evaluated from the same data set. The field visual group had the first turn median at 11.77 m (+2.12 m; −0.91 m) and the field “blind” group at 10.46 m (+0.91 m; −0.66 m). The field control group had their first turn median at 10.39 m (+0.67 m; −0.45 m). The search of the field visual group differed significantly from the searches of the two other groups (*p* < 0.01 against both other groups, ANOVA with Kruskal–Wallis post hoc test). **d** Two-dimensional search density plot of the field visual group. **e** Landmark alley seen from just above the nest entrance. **f** Two-dimensional search density plot of the field “blind” group. Details of the presentation are as in the previous figures

Ants that were trained and tested without manipulations, but with the alley of landmarks present (field visual group) (Fig. 4d, e), had a distance search centre at 10.90 m (*n* = 16). Ants that were trained and tested with the alley of landmarks present and with their ventral and lateral eyes covered (field “blind” group) (Fig. 4f) had a distance search centre at 9.59 m (*n* = 15). Ants that were trained and tested on a plain field without manipulations (field control group; compare Fig. 2f) had their distance search centre at 9.81 m (*n* = 21). The search of the field visual group differed significantly from the searches of the other groups (Fig. 4a, b) (*p* < 0.05 against field “blind” group, *p* < 0.01 against field control group, ANOVA with Holm-Sidak post hoc test). The search centre of the visual field group was thus shifted by about 12 % past the original feeding site position. Like in the channel experiments above, the situation was very similar when just the first turning points were analysed (Fig. 4c).

The shift in the search centres observed in both the channel visual group and the field visual group might have developed over time within a given search (see data for *Melophorus* in Schwarz et al. 2012). To scrutinise this possibility, the midpoints were calculated separately for the first and second, the third and fourth, and the fifth and sixth turning points in each individual, and compared statistically. There were no differences between the turning point medians in any of the groups, however, and the search distributions were clearly symmetric in the channel visual group (Fig. 3a, blue density distribution). Nor was there any discernible regression when performing correlation analyses or when applying Friedman’s test.

**Fig. 5:**
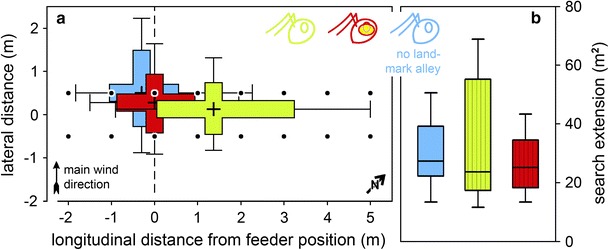
Search distribution characteristics (**a**), and search areas (**b**) of field searches. Details of the *box* plots are as in the previous figures, including *colour* identification of experimental groups. **a** For each individual ant, the search centres were calculated along the nest-feeder axis (abscissa, longitudinal distance) and along the axis perpendicular to it (ordinate, lateral distance) from all the *x* and *y* values in the search trajectory, including the *box*-and-*whisker* percentiles. Medians of all animals in an experimental group are shown to characterise the search distributions (e.g. the *whisker* at 5 m longitudinal distance represents the median of all 90 % percentiles in the field visual group). Note in the field visual group (*green*), the shift of search centre to 1.36 m (0.13 m lateral distance) along the landmark alley and the much increased longitudinal spread of the search distribution (3.21 m between 25 and 75 % percentiles, compared to 1.59 and 1.17 m in the field “blind” and the field control groups, respectively). Search width was reduced, by comparison (1.20 m between 25 and 75 % percentiles, compared to 1.36 and 1.78 m in the field “blind” and the field control groups). The landmark alley is drawn to *scale* on *top* of the search characteristics’ “*crosshairs*”. **b** Search areas were not altered by the presence of leading structures. *Vertically hatched boxes* indicate experiments with the landmark array present

The *searched area* was not significantly altered by the presence of leading structures (medians, field control group 27.3 m^2^, field visual group 23.7 m^2^, field “blind” group 25.2 m^2^) (Fig. 5b). However, the shape of the search distribution was distinctly extended along the alley of landmarks in the field visual group (Fig. 5a; see also density plot in Fig. 4d), at the expense of search width. The median value of search length in the field control group was 5.24 m, and the field visual group had a median search length of 7.59 m and the field “blind” group of 5.88 m. The search length of the field visual group is significantly different from the other groups (*p* < 0.01 vs. field control group, *p* < 0.05 vs. field “blind” group). The median width of search (determined orthogonal to the nest-feeder axis) in the field control group was 5.45 m; in the field visual group, it was 3.08 m and in the field “blind” group 3.91 m. Here, the field visual group differs significantly only from the field control group (*p* < 0.05).

In summary, the search characteristics of the field visual group were consistently different from the characteristics of the field control and field “blind” groups, with the notable exception of the searched area, as would be expected if visual perception of the leading structures indeed influenced search distributions.

## Discussion

In our experiments we reproduced the intriguing shift in food searches observed in *Cataglyphis* foragers that were familiar with a feeder located in a channel set-up (Bolek et al. 2012b) (Fig. 2e, f; grey box-and-whisker plots at bottom of each density plot). A shift in the food search away from the nest was observed not only in channels (Fig. 3) but also in the open desert terrain if the feeder was placed in an alley of landmarks extending from the nest past the feeding site (Fig. 4d, e). The shift in search behaviour was abolished in both situations by covering the animals’ ventral and lateral eye parts (Figs. 3, 4a, b, f), thus preventing visual recognition of channel walls and landmark alleys, respectively. These findings strongly suggest that the shift in search centre with experience is caused by visual cues, namely extended linear landmark structures such as the channel walls or the landmark alley. The desert ants apparently learn to use linear landmark arrays as leading structures when repeatedly visiting food sites. They use this knowledge to adjust their foraging behaviour in the sense that they concentrate the search to an area indicated by the leading structures and pay comparatively less attention to the distance indicated by their path integrator.

In the Australian desert ant *Melophorus bagoti*, gradual shifts in food (and nest) searches in the starting-point-to-goal direction were observed in channel experiments (Narendra et al. 2008; Schwarz et al. 2012). Such shifts occurred when the ants had learned with experience that food (or the nest) is located at the end of a channel (as it is often used in ant navigation research, e.g. Figs. 1a, 3, and Cheng and Wehner 2002). Guided by their path integrator, the animals started to search before they had reached the feeding site and shifted the search towards and even slightly past the food location if no food was encountered in the test situation. Apparently, in the constrained environment of a channel and in particular with the goal at the end of that channel, the ants learned to run along the channel, start a search just before the goal was to be expected and then mix this run-along-the-channel routine with their search programme. This resulted in a search gradually shifting along the channel since no channel end was encountered in the long test channel. It is important to note in this context that *Melophorus* typically lives in steppe-like habitats cluttered with grass tussocks and the occasional small tree. In this environment, *Melophorus* relies much more on panoramic and landmark information than on its path integrator (Narendra et al. 2008), although the path integrator is almost as reliable as in *Cataglyphis* (Cheng et al. 2014). *Melophorus* therefore usually does not run off its full path integration vector when steering towards a goal but rather relies on panoramic views and landmarks to pinpoint the target. If panoramic views and landmarks are absent, the Australian desert ant thus runs off only about half its vector before initiating a search (Narendra 2007), just as observed in the channel experiments outlined above. Intriguingly, the gradual shift in the starting-point-to-goal direction outlined above is less pronounced when the feeder is less conspicuously located on the side of the channel rather than at its end (Narendra et al. 2008; Schwarz et al. 2012). In this situation, *Melophorus* ants appear to rely more on their path integrator and less on learned landmarks, such as the channel end. In summary, actual search performance may depend on both (genetic) species differences and experimental protocols, even if the latter differ only in small details that are nonetheless important for the animals’ orientation.

The situation outlined for *Melophorus* is strongly reminiscent of the present results in *Cataglyphis*. However, *Cataglyphis*’ shift in search centre does not appear to develop over time, with subsequent search loops moving farther and farther away from the nest (Cheng and Wehner 2002). The search loops stay more or less focussed on the shifted search centre, and the search extended more along the guiding structures in the nest-feeder axis than perpendicular to it (Figs. 3d, 5b). We cannot strictly exclude such a shift with our present data set since we evaluated just six turning points. The gradual shift in *Melophorus* food searches develops over 10–18 turning points, although it is most pronounced during the initial six turns (Schwarz et al. 2012, their Fig. 4b). A gradual shift of *Cataglyphis’* search along the nest-feeder axis was never observed, though, neither in the present nor in any previous studies that evaluated ten turning points (e.g. Wittlinger et al. 2007a).

### Visual guidance cues in desert ants and other hymenoptera

Concerning the use of visual navigation cues, the above findings are in accord with the study of Wehner and coworkers (Wehner et al. 1996) who found that *Cataglyphis*’ lateral eye parts are necessary for landmark recognition. Knaden and coworkers (Knaden and Wehner 2005) observed that landmarks are able to shift search behaviour, especially if the home vector is close to the zero state. Apparently, such shifts occur also in food searches far away from the nest if the forager is on its outbound trip, searching for food. This has been demonstrated for *Melophorus* by a number of studies mentioned above (e.g. Cheng et al. 2009; Narendra et al. 2008; Schwarz et al. 2012); review in Cheng et al. 2014). In *Cataglyphis*, observations reminiscent of the present results were reported by Wolf and Wehner (Wolf and Wehner 2000, their Fig. 9) with regard to a displaced landmark array surrounding the feeder. With increasing experience, the ants heeded the feeder-defining landmark position more than the path integrator if these two cues were set in conflict.

It has been reported and indeed been analysed in considerable detail that (desert) ants use landmarks for orientation on their return to the nest. This holds for landmarks in the nest surrounds ((Åkesson and Wehner 2002; Bregy et al. 2008; Müller and Wehner 2010) see, e.g. (Collett 1995) for similar orientation strategies in wasps). It also holds for linear arrays or walls on the way home ((Collett et al. 1992, 1998, 2001) see, e.g. (Collett and Rees 1997; Collett et al. 2002) for similar strategies in bees and wasps). Such familiar landmarks may indeed elicit local vectors or motor programmes *en route* (Collett et al. 1992, 1998, 2001; Bisch-Knaden and Wehner 2003). The use of landmarks and extended structures during food searches has been comparatively little studied, (Wolf and Wehner 2000; Cheng and Wehner 2002; Graham and Collett 2002; Narendra et al. 2007, 2008; Schwarz et al. 2012; Schultheiss and Cheng 2013). It is perfectly clear, however, that insects may use landmark structure for guidance towards familiar feeding sites, particularly honeybees (review in (Collett and Collett 2002)). Intriguingly, shifts in search behaviour elicited by extended landmarks were not (yet?) observed in honeybees (see also article by Menzel and Greggers in this issue, addressing guiding structures in honeybee orientation). In *Cataglyphis*, the present observation of extended landmarks shaping search behaviour is not surprising, considering these animals’ versatile use of available orientation cues and the reports cited above. Food searches in ants deserve further scrutiny, however, because they differ from nest searches in a number of aspects. First, food searches usually have no definite goal, except when a feeding site has been identified as valuable by its food content and by experience (Wolf et al. 2012). Second, the motivations are clearly different, with the nest representing the primary location in an ant’s life for shelter and reproduction. The gradual shifts in food searches of Australian desert ants in channels (Narendra et al. 2008; Schwarz et al. 2012) provide an interesting example which illustrates that similar strategies may be used in food searches in different habitats and by different species, while there are significant differences in detail (Cheng et al. 2014). As noted above, experimental protocols may also have an influence here.

### Experience and weighting of different navigation mechanisms

In keeping with previous ideas, desert ants apparently possess a navigational toolkit from which they select the set of mechanisms that adequately accomplishes the navigational tasks at hand (Wehner 2009). In the present experiments, the ants’ path integrator is initially the dominant means of navigating back to a plentiful food source, centring the search on the previously visited location (Fig. 2e; Bolek et al. 2012b), in both channel and field experiments. With increasing experience, the different leading structures applied here increase in importance and thus shift the search along the leading structure if the food is not encountered at the familiar location (Fig. 2f, grey bottom box plot; Fig. 4a, d; compare also (Wolf and Wehner 2000), their Fig. 9). If no leading structures are present, the search remains centred on the previously successful site (Fig. 2b, d, f), which is also true if the guiding structures are rendered invisible by partial eye covers (Figs. 3, 4a, f). It should be interesting to examine more quantitatively how experience shapes the use of the different navigation tools over time (compare, e.g. Pelz et al. 1997 for honeybees). By the same token, it has to remain open on which navigation mechanism(s) the leading structures actually impinge. Several mechanisms work in parallel in ant navigation and apparently superimpose without cancelling each other out (Wehner 2009; Collett and Collett 2009; Collett 2012). For instance, the path integrator keeps running correctly even if the momentary navigation performance is dictated primarily by landmark cues (particularly illustrative example in (Andel and Wehner 2004). Effects of leading structures appear to exist with regard to route memory, motor learning, or the mode of landmark or panorama orientation (Collett et al. 1992, 1998, 2001; Bisch-Knaden and Wehner 2003; Collett and Collett 2009; Collett 2012).

A change in the relative weighting of navigation tools appears in accord with natural foraging situations of desert ants, in North Africa as well as in other desert biotopes (Cheng et al. 2006, 2009). In the desert, a given food source is usually soon exploited; in most instances, it is just a single insect carcass, and the forager has to search elsewhere next time. However, certain structures in the desert will produce increased food densities, for example, small shrubs and grass tussocks or even minor impressions in the desert floor. Wind-driven debris, including arthropod carcasses, will accumulate behind or inside such structures, making search behaviour along previously successful landmarks or floor structures useful indeed.

It is intriguing that, after a certain amount of training, the channel walls are used as leading structures in food search behaviour, whereas they appear not to be used in this way in *Cataglyphis’* nest search behaviour (this is different in *Melophorus*: Narendra et al. 2008; Schwarz et al. 2012). This is implicit in a large number of navigation studies in channels that have employed nest search as experimental paradigm (e.g. in (Cheng and Wehner 2002; Wittlinger et al. 2006, 2007a, b), and it is explicitly addressed by Merkle and Wehner (Merkle and Wehner 2009)). Deviations from the expected search distance observed in nest search behaviour are usually small, if present at all. The prominent and well-studied examples were observed in particular situations and are due to leaky integrator (Sommer and Wehner 2004) or approximation (Müller and Wehner 1988) properties of the path integrator. This indicates that even though the basic principles of search behaviour (Müller and Wehner 1994) remain the same, the strategies may differ with regard to the inclusion of additional cues such as (extended) landmarks, an aspect that warrants future scrutiny. As indicated above, the situation is different in the Australian desert ant *Melophorus*, where leading structures are also used in nest searches (Narendra et al. 2008; Schwarz et al. 2012), primarily due to the abundance of landmarks and panorama cues in the habitat.

### Visual properties of leading structures

There were a number of noteworthy further observations in our study. First, preliminary experiments showed that the eye covers in the “blind” groups had to extend further dorsally in the open desert terrain than in the channel experiments to completely abolish landmark recognition. We thus decided to apply the eye covers always up to the dorsal rim area. This observation is intriguing since the landmarks in the alley of black cylinders cover less of the ants’ field of view than do the channel walls (from the centres of the leading structures, the tops of the landmarks appear at an angle of about 31°, whereas the upper edges of the channel walls appear at an angle of about 63°). While the black landmarks and the grey channel wall should provide the same (green) contrast against the sky for the ant visual system (e.g. Graham and Cheng 2009; Möller 2002; Philippides et al. 2011), the landmark alley is more salient due to the repetitive landmark arrangement. The channel walls are straight and without salient features, effectively representing an elevated straight horizon. The landmark alley thus appears to represent a stronger visual stimulus than the homogeneous grey channel. This may be the reason why a larger eye area has to be covered in the landmark alley to sufficiently exclude visual perception of this leading structure. This observation corroborates the statement from above that details of the experimental protocol may produce (unexpected) differences in behavioural performance.

The fact that channels and landmark alleys have similar effects on food search behaviour as leading structures raises an intriguing question. Desert ants are able to use landmarks to pinpoint familiar feeding sites (Wolf and Wehner 2000), like many other insects (e.g. Collett and Rees 1997). Under which conditions are the landmarks in the alley used as actual landmarks instead of, or in addition to, their use as leading structures? The one-dimensional leading structure extends search behaviour longitudinally, leading to an increased probability of finding food along the leading structure, as derived from previous experience. A landmark array around the feeder, namely the set of landmarks in the alley that is closest to the feeder, should concentrate the search on the feeding site (Wolf and Wehner 2000), by contrast. A change between the two orientation strategies should be clearly discernible in experiments using different gaps between the landmarks in the alley or other suitable arrangements. In keeping with this idea, there is a clear second focus of search density in the landmark alley at 11 m, that is, one pair of landmarks past the original feeder location (Fig. 4a; second green arrow), and there are shoulders in the search distribution at 13 and 15 m. These peaks and shoulders probably indicate search concentrations elicited by the landmarks themselves, rather than by their arrangement into an alley. Nonetheless, the effect of the alley in shifting the search past the original feeder position is evident from the shifted search density distribution (Fig. 4d).

### Organisation of search behaviour

It was another interesting result that the search of the ants covered virtually the same area in the field visual group and the other groups (Fig. 5b). The latter groups, field “blind” and control groups, did not exhibit the elongation of search distribution along the leading structure (Fig. 4f) typical of the channel and landmark alley searches. Usually, the search is centred on a certain point (Müller and Wehner 1994), resulting in an approximately circular search area (Figs. 2f, 4f). In the field visual group (Fig. 4a, d), the ants increased the length of the searched area while reducing the width accordingly. They, thus, adapted the shape of the search area according to the landmark array, preferring one axis for the search (compare Biegler 2000; Wolf and Wehner 2005) and thus forming an oblong (Figs. 4d, 5a). This indicates that the ants search a given area, the size of which is independent of the shape of the searched space. The size of the searched area rather appears to depend on the motivation of the foragers, that is, the number of training visits to the feeder and the food available in the feeder (Bolek et al. 2012b; Wolf et al. 2012), or on the uncertainty about the goal position (e.g. Fig. 3.35 in Wehner 1992, 2009; Wolf and Wehner 2005; Merkle and Wehner 2010). This would appear to improve search efficiency by concentrating the search to an area with a higher probability of success, in our case between the rows of the landmark alley.
